# Optimizing operational parameters for the enzymatic production of furandicarboxylic acid building block

**DOI:** 10.1186/s12934-021-01669-1

**Published:** 2021-09-09

**Authors:** María Isabel Sánchez-Ruiz, Angel T. Martínez, Ana Serrano

**Affiliations:** Centro de Investigaciones Biológicas “Margarita Salas” (CIB), CSIC, Ramiro de Maeztu 9, 28040 Madrid, Spain

**Keywords:** Enzymatic catalysis, Hydroxymethylfurfural oxidase, Catalase, Enzyme engineering, 2,5-Furandicarboxylic acid product, 5-Formylfurancarboxylic acid intermediate, Reaction pH, Hydrogen peroxide by-product, Enzyme inhibition, Reaction optimization

## Abstract

**Background:**

2,5-Furandicarboxylic acid (FDCA) is a precursor for green plastics due to its structural similarity to terephthalic acid, a common precursor of oil-derived polymers, and its potential production from sugars obtained from plant biomass. Hydroxymethylfurfural oxidase (HMFO) has been reported as a promising biocatalyst for FDCA production since it can convert bio-based 5-hydroxymethylfurfural (HMF) into FDCA building block. This three-step oxidation reaction occurs through the diformylfuran and 2,5-formylfurancarboxylic acid (FFCA) intermediates. Several efforts have been made for the development of HMFO variants that increase FDCA yields by improving their activities over the reaction intermediates. However, there is still limited insight into how operational conditions can influence these enzymatic reactions. The setup of optimal reaction conditions would enable to understand potential problems hampering the effective industrial production of this bioplastic precursor using HMFO as biocatalyst.

**Results:**

In this work, several parameters affecting the performance of *Methylovorus* sp HMFO oxidizing HMF have been analyzed for the wild-type enzyme, and its V367R and W466F single variants, V367R/W466F double variant, and I73V/H74Y/G356H/V367R/T414K/A419Y/A435E/W466F (8BxHMFO) octuple variant. Our results show how the oxidation of HMF by HMFO enzymes is highly influenced by pH, with different optimal pH values for the different improved variants. Moreover, the enzymes are not stable at high hydrogen peroxide concentrations and their activity is inhibited by the FFCA intermediate in a pH-dependent way. These limitations can be efficiently overcome with the addition of catalase to the reaction medium, which removes the hydrogen peroxide formed during the oxidations, and the controlled dosage of the substrate to limit the amount of FFCA accumulated in the reaction. The different behavior of wild-type HMFO and its variants against pH, hydrogen peroxide and FFCA highlights the importance of considering each variant as an individual enzyme with its own operational conditions for an eventual industrial FDCA production.

**Conclusions:**

This work provides information of those parameters that condition a high production of FDCA by HMFO. Unraveling these factors allowed to increase the FDCA yields by using the most stable enzymes at their optimal pH for HMF oxidation, removing the peroxide with catalase, and avoiding FFCA accumulation by controlling substrate and/or enzyme concentration. These above findings will be useful when planning a future scale-up of these conversions and will provide new viewpoints for the design of HMFO variants that render a more effective performance during HMF conversion into FDCA.

**Supplementary Information:**

The online version contains supplementary material available at 10.1186/s12934-021-01669-1.

## Background

A 2017 global analysis estimated in over 8000 million tons the total amount of virgin plastics produced by the petrochemical industry [1]. This production generated high greenhouse gas emissions from non-renewable fossil resources, and caused both land and water pollution due to limited recycling. Substitution of these oil-based plastics with biodegradable polymers derived from renewable raw materials is thus necessary for the development of a sustainable bio-economy [2]. One of the most promising bio-based plastic polymers is poly(ethylene-2,5-furandicarboxylate) (PEF) [3, 4]. PEF can be used as a green substitute for conventional poly(ethylene-terephthalate) (PET) due to its similar, or even better, properties [5, 6]. The interest of PEF lies in its composition, as it is formed by esterification of ethylene glycol with the renewable building block 2,5-furandicarboxylic acid (FDCA), which can be obtained from sugars derived from lignocellulosic biomass [7, 8].

**Fig. 1:**
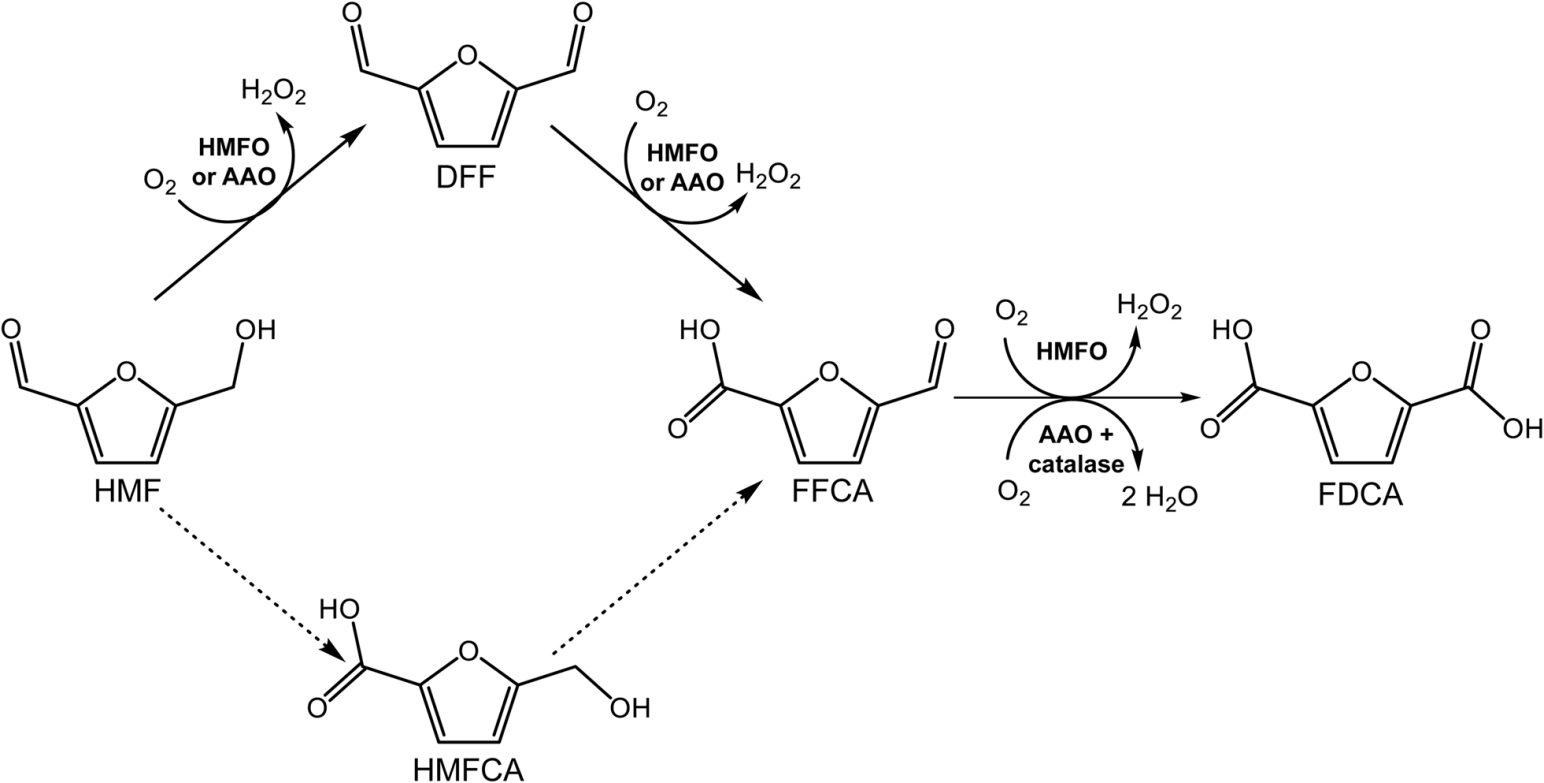
5-Hydroxymethylfurfural (HMF) conversion into 2,5-furandicarboxylic acid (FDCA). Possible routes, via 2,5-diformylfuran (DFF) or 2,5-hydroxy-methylfurancarboxylic acid (HMFCA) intermediates, to obtain 2,5-formylfurancarboxylic acid (FFCA) and FDCA are shown. Reactions catalyzed by hydroxymethylfurfural oxidase (HMFO) and by aryl-alcohol oxidase (AAO) are indicated with full arrows. FFCA oxidation by AAO in combination with catalase (decomposing the H_2_O_2_ generated during the reaction) is also shown [25]

The processes for FDCA production from lignocellulosic feedstocks usually comprise two steps. First, monosaccharides (generally fructose) derived from plant polysaccharides are dehydrated to form the platform chemical 5-hydroxymethylfurfural (HMF). Second, HMF is converted into FDCA through three consecutive oxidation steps, involving its alcohol and aldehyde groups, that firstly yield diformylfuran (DFF) or hydroxymethylfurancarboxylic acid (HMFCA), respectively, and then formylfurancarboxylic acid (FFCA) as reaction intermediates (Fig. 1) [9, 10]. Different chemical methods have been described for HMF conversion into FDCA, however, they typically lead to low yields and selectivities, and require high temperatures and pressures and the use of metal salts and organic solvents that render the process expensive and polluting [11, 12]. Thus, in the context of green industry, biocatalytic alternatives to these processes are highly interesting, since the reactions can be performed under environmentally-friendly conditions using mild and biodegradable catalysts [13].

A variety of studies have been performed for the production of FDCA using both enzymatic and whole-cell catalysis [8, 14, 15]. With this aim, several oxidative enzymes have been suggested for the production of FDCA from HMF. However, because most of them are limited to either aldehyde or alcohol oxidation, multi-enzymatic cascades are usually required to complete the reaction [16–22]. This often limits the overall yield of the process since a consensus between the operational conditions of each enzyme needs to be established. Up to now, only two enzyme types—hydroxymethylfurfural oxidase (HMFO) from *Methylovorus* sp MP688 [23] and *Pseudomonas* species [24], and aryl-alcohol oxidase (AAO) from *Pleurotus eryngii* [25]—have been reported to carry out the three oxidation steps for HMF conversion into FDCA. Both are FAD-containing enzymes of the glucose-methanol-choline oxidase/dehydrogenase (GMC) superfamily [26] with activity on both primary aryl alcohols and hydrated aryl aldehydes. Due to their preference for alcohol substrates, they oxidize HMF into FDCA through the DFF and FFCA intermediates (Fig. 1). However, several characteristics would confer HMFO advantages for the industrial production of FDCA, compared to AAO. First, the bacterial HMFO can be easily overexpressed in *Escherichia coli* in a soluble active form [24, 27], in contrast to fungal AAO that is typically produced as inclusion bodies in *E. coli* hosts and thus requires in vitro activation [28] (a time and money consuming process). Nevertheless, the soluble production of *P. eryngii* AAO using *Komagataella pastoris* (syn. *Pichia pastoris*) has been recently reported [29], although a more detailed analysis of the effect of its hyper-glycosylation on furfural oxidation is required. Second, the oxidation of FFCA, which constitutes the limiting step for the enzymatic production of FDCA from HMF, is more efficient for HMFO, since FFCA oxidation by AAO requires long reaction times and is highly inhibited by H_2_O_2_ [25]. In addition, HMFO production has been recently upscaled in a cost-effective bioreactor process [30], a crucial factor when considering its industrial application as a biocatalyst.

Due to its promising use for FDCA production, structural and mutagenic studies of the active site of *Methylovorus* HMFO by Dijkman et al*.* [31] led to identify the V367R, W466F and V367R/W466F variants as improved biocatalysts. These engineered enzymes perform a faster oxidation of FFCA, the rate-limiting reaction in HMF conversion, than the wild type (WT) enzyme. Moreover, by combining computational prediction and gene shuffling approaches, a multiple variant (8BxHMFO) with eight mutations (I73V/H74Y/G356H/V367R/T414K/A419Y/A435E/W466F) was designed in subsequent studies as a more robust and stable enzyme during catalysis [32]. However, studies on the operational conditions for HMF oxidation into FDCA (using the above HMFO WT and derived variants) are scarce, and a focused analysis of their behavior during these conversions is needed before proposing them as industrial biocatalysts.

In this work, a comprehensive study on different parameters that could affect the performance of *Methylovorus* HMFO in the production of FDCA from HMF has been carried out for HMFO WT and its V367R, W466F, V367R/W466F and 8BxHMFO variants. Thus, the effect of pH on enzyme stability and activity, the inhibition by reaction product and by-product, the initial substrate concentration, the cofactor dependency, and the oxygen reactivity have been evaluated. This allowed to optimize the operational conditions for each enzyme variant, and to bring closer the application of the most relevant candidates for the enzymatic production of FDCA at an industrial scale. The information obtained will provide useful insights for future studies with HMFO-type enzymes, and will also contribute to the development of new HMFO variants for enhanced FDCA production from HMF.

## Results and discussion

### Effect of pH on FDCA production

**Fig. 2:**
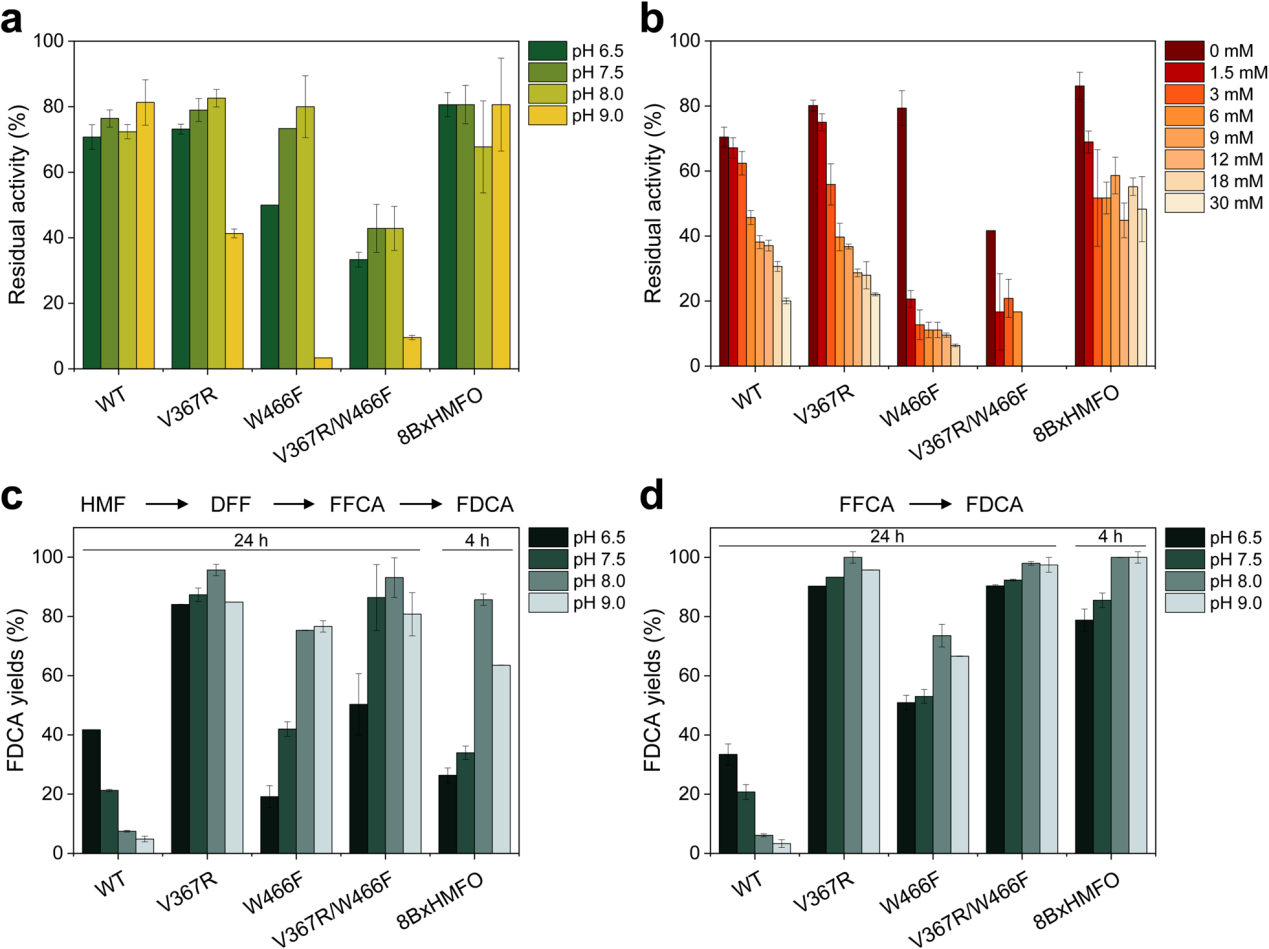
Effect of pH (**a**) and H_2_O_2_ (**b**) on HMFO stability, and influence of pH on FDCA production from HMF (**c**) and FFCA (**d**). **a** pH stability as shown by residual activities after 72-h incubation of the enzymes in 100 mM B&R buffer (pH 6.5–9) at 25 °C. **b** H_2_O_2_ stability as shown by residual activities after 72-h incubation of the enzymes with increasing amounts of H_2_O_2_ (0–30 mM) in 50 mM Tris/HCl, pH 7.5. The residual activities were measured with vanillyl alcohol as substrate. Percentages of FDCA (referred to initial substrate concentration) in **c** and **d** are shown after 24-h reaction of HMF or FFCA, respectively, with the HMFO WT and variants (4-h reaction with 8BxHMFO) at different pH values. The reactions were performed at 28 °C and 180 rpm shaking, using 1.5 mM substrate, and 2.5 µM enzyme, in 50 mM NaPi (pH 6.5) or Tris/HCl (pH 7.5, 8.0 and 9.0). Mean and standard deviation values from triplicate experiments are shown

First, the pH stability of the enzymes was analyzed by measuring their residual activity (on the standard substrate vanillyl alcohol) after 72 h of incubation in the range of pH 6.5–9.0 (Fig. 2a) since it has been reported that the *Methylovorus* enzyme and other HMFOs totally lost their activity after 24 h out of this range [24]. HMFO WT and 8BxHMFO remained stable in the full pH range, keeping 70–80% of their initial activity. V367R retained ~ 80% of its activity between pH 6.5 and 8.0, and ~ 40% at pH 9.0. Similar behaviors were observed for the W466F and V367R/W466F variants, as both remained more active at pH 7.5–8.0, but they lost most of the activity at pH 9.0. The V367R/W466F variant showed to be particularly unstable, as it lost > 50% of its activity at all the pH values tested.

A high dependence on pH has been recently reported for FDCA production by a WT HMFO [24]. Thus, this possible effect was evaluated here for the different variants. With this purpose, 24-h enzymatic reactions were performed at 28 °C in 50 mM NaPi, pH 6.5, or in 50 mM Tris/HCl, pH 7.5–9.0, and the production of FDCA from 1.5 mM HMF or FFCA was analyzed by high-performance liquid chromatography (HPLC) after 24 h (Fig. 2c and d, respectively). For the 8BxHMFO variant, yields after 4-h reaction were considered, since at 24 h total conversion was observed at all the pH values. For HMFO WT the optimal pH for FDCA production was 6.5. Surprisingly, all HMFO variants shifted this optimum to pH 8.0–9.0 for the oxidation of both substrates. The pH values in which the maximal half-lives were observed during HMF and FFCA conversion (Table 1) coincided with the pH at which each enzyme achieved the highest FDCA yields in 24-h reactions (Fig. 2c and d).

**Table 1:** Half-lives (h) of HMFO WT and variants (2.5 µM) during FDCA production from HMF and FFCA (1.5 mM) in 48-h reactions, at different pH values

Enzyme	Substrate	pH 6.5	pH 7.5	pH 8.0	pH 9.0
WT	HMF	**85.6**	52.5	23.0	28.5
V367R		7.0	24.8	**27.8**	6.8
W466F		3.9	8.5	**8.7**	3.9
V367R/W466F		5.7	13.0	**18.8**	13.7
8BxHMFO		34.1	36.1	**144.0**	24.4
WT	FFCA	**204.0**	187.0	114.0	112.0
V367R		60.3	61.9	**97.6**	105.0
W466F		18.4	26.5	**40.5**	38.7
V367R/W466F		34.1	41.5	**68.6**	16.9
8BxHMFO		142.0	204.0	**347.0**	204.0

Residual activities were measured with vanillyl alcohol in 50 mM Tris/HCl, pH 7.5. The highest value for each enzyme is shown in bold

### Effect of H_2_O_2_ byproduct on FDCA production

In the oxidation of HMF to FDCA, three equivalents of H_2_O_2_ are produced by HMFO since the reaction takes place through two furfural intermediates (Fig. 1). Thus, another aspect to be considered is how this H_2_O_2_ would affect the enzyme stability or the reaction itself.

To study if the H_2_O_2_ accumulated during the reaction had some negative effect in enzyme stability, HMFO WT and its variants were incubated for 72 h with 0–30 mM H_2_O_2_ in Tris/HCl, pH 7.5, at 25 °C, and their residual activities were measured with vanillyl alcohol (Fig. 2b). All the enzymes were affected by H_2_O_2_ with the W466F and V367R/W466F variants retaining < 20% of the initial activity at all the H_2_O_2_ concentrations assayed. In contrast, the 8BxHMFO multiple variant kept at least ~ 50% of its activity in the whole H_2_O_2_ range, while the activity of the HMFO WT and V367R variant was always below 50% at H_2_O_2_ concentrations higher than 3 mM.

To discard an additional inhibitory effect of H_2_O_2_ on the FFCA oxidation reaction, as reported for AAO [25], FDCA production from FFCA by HMFO WT was evaluated along 48 h in the presence of 1.5–18 mM H_2_O_2_ (Additional file 1: Fig. S1). However, no strong differences were observed compared with the FDCA produced in absence of external H_2_O_2_. The H_2_O_2_ generated during catalysis was removed by the addition of catalase to increase the stability of the enzymes. The effect of catalase was milder at low substrate concentrations (~ 1.5 mM) but it was more relevant at higher HMF concentrations, as described below.

**Fig. 3:**
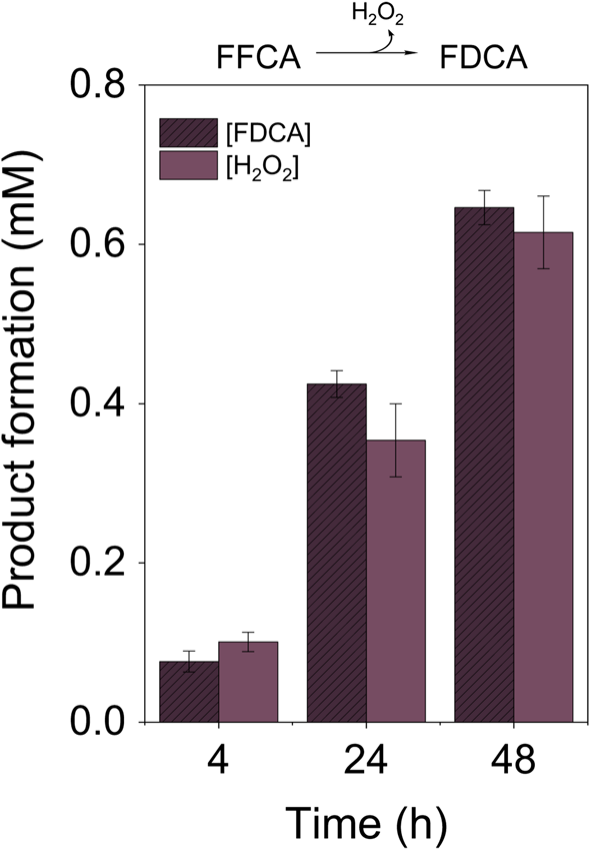
Comparison of H_2_O_2_ and FDCA production during FFCA oxidation by HMFO. Reactions were performed at 28 °C in 50 mM Tris/HCl, pH 7.5, between FFCA (1.5 mM) and HMFO WT (2.5 µM). The products, FDCA and H_2_O_2_, were quantified after separation of the reaction mixture by HPLC and with AmplexRed/HRP assay, respectively. Mean and standard deviation values are shown

The H_2_O_2_ produced by HMFO along the reaction with FFCA was determined with the AmplexRed®/horseradish peroxidase (HRP) coupled assay, and compared with the amount of FDCA quantified by HPLC (Fig. 3). The results showed stoichiometric amounts of FDCA and H_2_O_2_ along the reaction, indicating that the oxidation of FFCA by HMFO takes place with H_2_O_2_ release. This contrast with the slower catalysis by AAO that, given the lack of H_2_O_2_ formation, was proposed to occur through a monooxygenase-type mechanism [25]. The difference makes the oxidation of FFCA by HMFO more efficient than by AAO, which requires much longer reaction times.

### Effect of FFCA intermediate on FDCA production

**Fig. 4:**
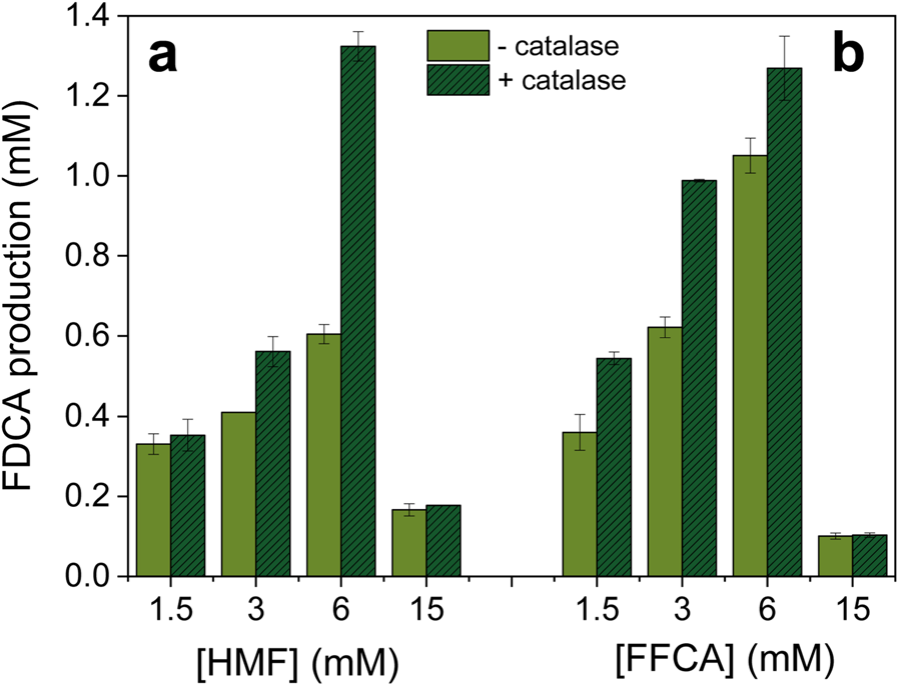
FDCA production from different HMF (**a**) and FFCA (**b**) concentrations and catalase effect. FDCA production by HMFO WT (2.5 µM) from increasing HMF (left) and FFCA (right) concentrations (1.5–15 mM) is shown in the absence and presence of catalase. Reactions (48 h) were performed in 50 mM Tris/HCl, pH 7.5, at 28 °C. Mean and standard deviation values are shown

Although HMFO is capable to carry out HMF oxidation to FDCA, its lower activity towards FFCA limits the efficiency of the whole reaction [23, 24]. To displace the reaction towards product formation we evaluated the effect that increasing amounts of HMF or FFCA had on FDCA production by HMFO WT (Fig. 4a and b, respectively). At the highest substrate concentrations assayed (15 mM) the FDCA yields from HMF decreased drastically. The fact that similar inhibition was observed with increasing amounts of FFCA suggests that the reaction is inhibited by this reaction intermediate. In the presence of catalase, the same inhibition profiles were observed upon increasing substrate concentration, although higher FDCA yields were attained, being the highest with 6 mM HMF (Fig. 4a).

**Fig. 5:**
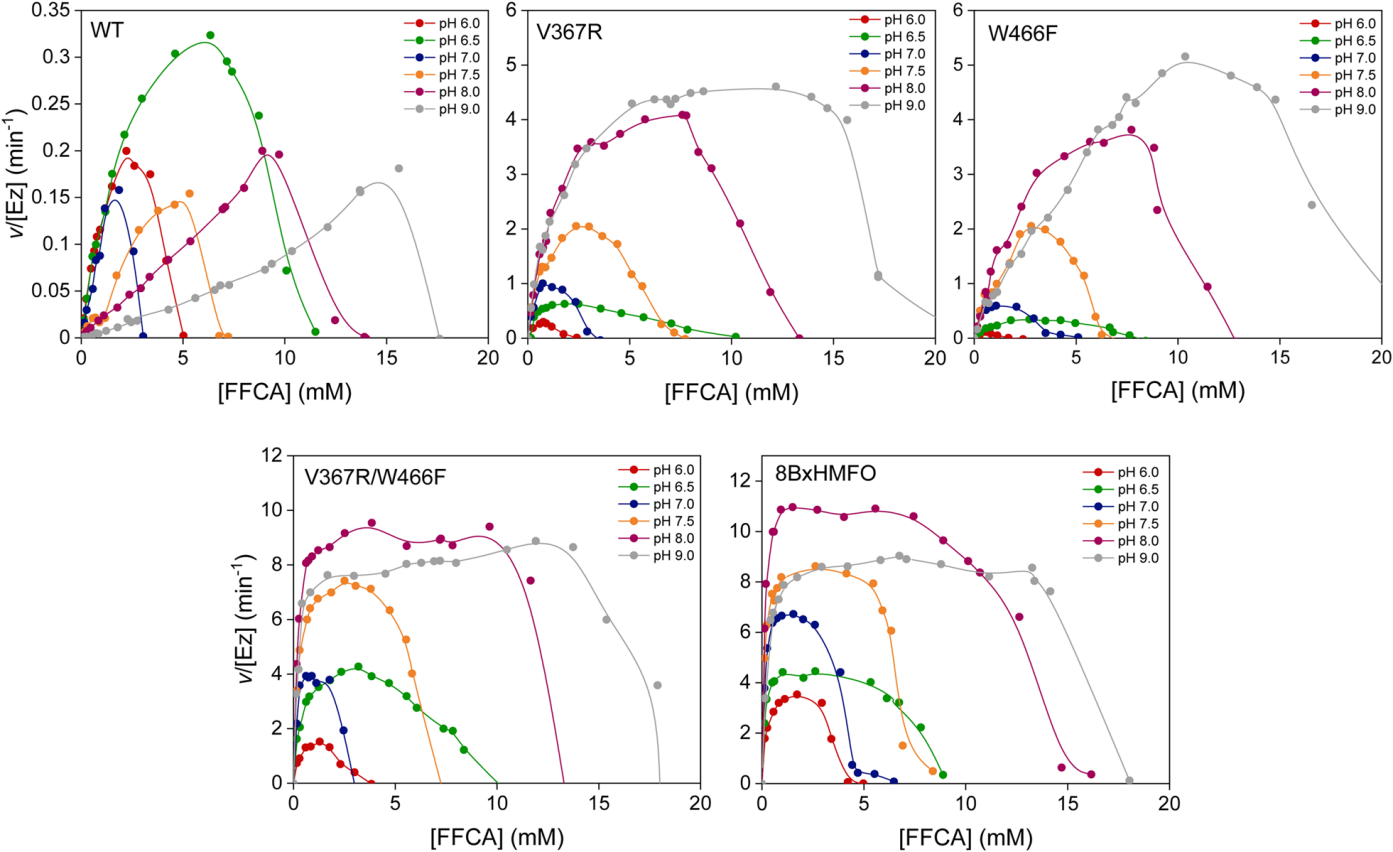
Effect of pH on kinetic curves of FFCA oxidation by HMFO WT and variants. Reaction rates (*v*/[Ez] as µmol of product per µmol of enzyme per time, in min^−1^) were obtained for end-point reactions of HMFO WT, and its V367R, W466F, V367R/W466F and 8BxHMFO variants with increasing amounts of FFCA, in 50 mM NaPi (pH 6.0–6.5) or Tris/HCl (pH 7.0–9.0) at 25 °C, in the presence of catalase. FDCA production was quantified in 48-h and 30-min reactions for the HMFO WT and variants, respectively

The steady-state kinetics for HMF and DFF oxidations were measured with AmplexRed®/HRP in a continuous assay. The saturation profiles for all the variants discarded any inhibitory effect by these two substrates (Additional file 1: Fig. S2 and Table S1). However, an end-point method was required to analyze the kinetics for FFCA oxidation, given the much lower activities on this substrate. Since important differences in FDCA yields from FFCA were observed depending on pH, these kinetic measurements were performed at pH values from 6.0 to 9.0, and an excess of catalase was added to ensure the enzyme stability. Reactions were stopped after 48 h for HMFO WT and after 30 min for the improved HMFO variants, and the FDCA formed was quantified by HPLC (Fig. 5). Both HMFO WT and variants showed a strong inhibition by FFCA that, surprisingly, was highly dependent on pH. To better understand this inhibition, residual activity of HMFO WT was measured (with vanillyl alcohol) when FFCA was added (time 0 h) and before stopping the reaction after 48 h, at the different substrate concentrations used in the kinetic curves (Additional file 1: Fig. S3a and b). The results indicate that at those FFCA concentrations where inhibition was detected, the enzymatic activity was lost from the moment that enzyme and FFCA were put together. To know if this inhibition could be reverted, enzymes were dialyzed in 50 mM Tris/HCl overnight, pH 7.5, after their incubation with 15 mM FFCA for 5 min. However, all of them remained inactive (data not shown) indicating irreversible inhibition by FFCA. These data suggest that FFCA would bind at the active site or in its access channel preventing the enzyme to act in successive catalytic cycles.

Due to the high inhibition observed at different pH values (Fig. 5), only apparent kinetic constants for FFCA could be calculated for each enzyme and pH (Table 2). Catalytic efficiencies were the highest at pH 6.0 for HMFO WT and in the range of pH 7.0–8.0 for the different variants. For all the enzymes, inhibition occurred at higher substrate concentrations when kinetics were performed at more basic pH. Moreover, 8BxHMFO showed better catalytic efficiencies than V367R/W466F and the other variants at any pH tested. This pH-dependent inhibition by FFCA is in agreement with the efficiencies observed at the different pH values in long-term HMF and FFCA reactions (Fig. 2c and d), being the optimal pH for these conversions those in which the enzymes present less FFCA inhibition (within the pH range in which the highest apparent efficiencies are observed).

**Table 2:** Apparent catalytic efficiencies (*k*_cat_/*K*_m_, mM^−1^ min^−1^) for FFCA oxidation by HMFO WT and variants, at different pH values

pH	WT^a^	V367R	W466F^a^	V367R/W466F	8BxHMFO
6.0	0.24 ± 0.04	1.6 ± 0.6	0.21 ± 0.01	8.2 ± 2.2	24.9 ± 4.3
6.5	0.18 ± 0.01	3. 0 ± 0.2	0.55 ± 0.10	13.6 ± 1.4	51.6 ± 11.1
7.0	0.10 ± 0.01	4.9 ± 1.7	1.84 ± 0.32	42.3 ± 14.4	76.8 ± 11.2
7.5	0.033 ± 0.001	4.1 ± 0.7	1.65 ± 0.24	43.2 ± 2.6	95.9 ± 12.2
8.0	0.023 ± 0.001	3.7 ± 0.3	1.84 ± 0.32	66.4 ± 7.3	130.0 ± 16.0
9.0	0.009 ± 0.001	3.2 ± 0.3	1.65 ± 0.24	44.8 ± 5.2	45.1 ± 2.9

^a^For HMFO WT in pH ≥ 7 and for W466F in all the pH range, the efficiencies were estimated as *k*_obs_/[FFCA] ratios due to the lack of enzyme saturation (before its inhibition). Mean and standard deviation values are shown

### Effect of O_2_ concentration on FDCA production

The high enzyme inhibition by FFCA prevented evaluation of the reaction under saturating HMF concentrations. However, we were able to evaluate the effect of oxygen saturation (the second substrate required by the oxidase) on FDCA production by HMFO WT and its variants. With this purpose, the enzymes were mixed with 6 mM HMF and catalase excess at 28 °C, bubbled with O_2_, corresponding to 1.22 mM concentration [33], and the FDCA yields were compared with those obtained using atmospheric O_2_, corresponding to 0.25 mM concentration, after 48-h reaction (Additional file 1: Fig S4a). Although in the case of HMFO WT some improvement was observed at higher O_2_ concentration, for the HMFO variants, which are more efficient for FFCA oxidation, similar results were obtained under both conditions. This suggests that the current reactions would be already saturated under atmospheric O_2_. Nevertheless, additional improvements enabling use of higher substrate concentrations, an important point for scaling up the process, could be obtained by increasing the O_2_ diffusion into the reaction medium. In this sense, continuous-flow microreactor technology has been reported as a safe and scalable way to approach oxidation reactions [34, 35] and different reactor designs—such as simple flow reactors, tube-in-tube reactors, agitated tube reactors and continuous agitated cell reactors—have been used for O_2_-dependent enzymes showing higher oxygen transfer rates than in batch reactions [36–40].

### Effect of FAD addition on FDCA production

It has been suggested that higher FDCA yields can be attained by adding FAD to HMFO WT reactions [23] to prevent eventual inactivation of the enzyme by cofactor dissociation. Although such effect was claimed based on 95% FDCA yield in 24-h reactions of 4 mM HMF (and 20 µM HMFO) in presence of 20 µM FAD, the study lacked the FAD-less controls (with 4 mM HMF and 20 μM HMFO) to demonstrate such effect. Therefore, to confirm/discard a positive effect of FAD, HMF (6 mM) reactions with and without 20 μM FAD, in pH 6.5 50 mM NaPi for HMFO WT and pH 8.0 Tris/HCl for the variants, were performed here. The FDCA yields and residual activities of the enzymes (enabling half-life, t_1/2_, calculation) were followed along 48 h (Table 3 and Additional file 1: Fig. S4b). No differences were observed for HMFO WT and V367R, while a slight increase was observed when FAD was added to variants bearing the W166F mutation (~ 1.1-fold for 8BxHMFO and 1.5 fold for W466F and V367R/W466F). Since the efficiency for FFCA oxidation was similar in absence and presence of FAD (Additional file 1: Table S2) the slight effect of the cofactor can be attributed to higher stability of these variants when FAD was added to the reaction (with half-lives 1.2–1.5-folds higher than in absence of the cofactor). These results make sense for variants containing a mutation at W466, since this residue is close to the flavin ring, and its removal affect protein stability [31].

**Table 3:** Effect of FAD or/and catalase on catalytic performance parameters—FDCA concentration (mM) and yield (%), enzyme half-life (t_1/2_), and total turnover number (TTN)—for the production of FDCA from HMF (6 mM) by the HMFO WT and variants

Enzyme	FAD	Catalase	FDCA (mM)	FDCA (%)	t_1/2_ (h)	TTN
WT	−	−	1.4	23	16	1650
	+	−	1.4	23	17	1680
	–	+	2.1	35	178	2520
	+	+	3.1	53	210	3810
V367R	–	−	3.5	58	19	4200
	+	−	−	59	18	4240
	–	+	6.0	100	128	7200
	+	+	6.0	100	315	7200
W466F	−	−	−	8	23	605
	+	−	−	12	27	880
	−	−	4.8	80	35	5730
	+	+	4.0	67	49	4820
V367R/W466F	−	−	−	24	24	1740
	+	−	−	37	34	2650
	−	−	4.3	74	64	5350
	+	+	3.2	53	76	3800
8BxHMFO	−	−	−	87	26	6240
	+	−	−	96	38	6900
	−	−	6.0	100	204	7200
	+	+	6.0	100	315	7200

Reactions were performed using 6 mM HMF and 2.5 µM enzyme in 50 mM NaPi, pH 6.5 (for HMFO WT) and 50 mM Tris/HCl, pH 8.0 (for the HMFO variants), at 28 °C. 20 µM FAD and/or catalase excess were added, when indicated

Anyway, it is necessary to remark that the effect of FAD is much lower than the effect of adding catalase to the reaction (Table 3 and Additional file 1: Fig. S4b). The removal of H_2_O_2_ by catalase positively affects both stabilities and FDCA yields for all the enzymes. An improvement of 1.5–3-folds was observed for most of the variants and up to 9-folds for W466F, while the half-lives significantly increased for HMFO WT and the V367R and 8BxHMFO variants. In these conditions, variants V367R and 8BxHMFO achieved full conversion of 6 mM HMF into FDCA after 48 h of reaction. Moreover, although the addition of catalase plus FAD increased the half-life of all the enzymes, the FDCA yield was only increased for HMFO WT (while FDCA production by its variants was similar to that observed upon the only addition of catalase). Finally, it was confirmed that only traces of HMFCA (< 1%) were produced in HMF controls with and without catalase or FAD in the absence of enzyme, excluding any activity of catalase or FAD oxidizing these furfurals (Additional file 1: Fig. S5).

### Optimized conditions for FDCA production

Taking all the above results together, the conditions for FDCA production with the HMFO enzymes were optimized. Among the variants analyzed, V367R and 8BxHMFO were selected as the most suitable for the scalability of the process, since they exhibit higher stability and better performance. Reactions were carried out at pH 8.0 (in 50 mM Tris/HCl) since this is the optimal pH for FDCA production by these variants (Fig. 2). The stability of the enzymes during the reactions was ensured by addition of a catalase excess, as shown by their higher half-life under these conditions (Table 3). The addition of FAD was excluded since the benefit is not relevant, as shown above, and the high cost of the cofactor limits its use for an industrial application.

Considering the relatively low substrate concentration used in the previous experiments, we explored increasing HMF concentrations (up to 12 mM, since accumulation of higher concentrations of FFCA results in enzyme inhibition, Fig. 5). However, the final amounts of FDCA product were barely improved, reaching < 7.5 mM of FDCA after 6 days of reaction, despite of the use of higher HMF concentration (Additional file 1: Fig. S6a). Moreover, under these conditions, the stability of the enzymes decreased drastically over time (Table 4 and Additional file 1: Fig. S6b).

**Table 4:** Catalytic performance parameters—FDCA concentration (mM) and yield (%), residual activity, and total turnover number (TTN)—for the production of FDCA from 6–12 mM HMF (including 6 mM re-dosage after 6 mM initial concentration) by the V367R and 8BxHMFO variants

Enzyme	HMF (mM)	Enzyme (µM)	FDCA (mM)	FDCA (%)	Residual activity (%)	TTN
V367R	6	2.50	6.0	100	67	7200
	8	2.50	7.0	88	0	8460
	10	2.50	5.0	50	0	6025
	12	2.50	2.0	17	0	2376
	6 + 6^a^	2.50	9.3	78	2	11,200
	6 + 6^b^	1.25	11.7	98	3	27,400
8BxHMFO	6	2.50	6.0	100	75	7200
	8	2.50	7.4	92	10	8830
	10	2.50	6.1	60	8	7204
	12	2.50	5.3	45	4	6500
	6 + 6^a^	2.50	9.6	80	37	11,600
	6 + 6^b^	1.25	10.7	89	36	26,200

Reactions were performed in 50 mM Tris/HCl, pH 8.0, for 6 days (12 days when the enzyme concentration was reduced to 1.25 µM) in the presence of catalase excess. The residual HMFO activity was estimated with vanillyl alcohol, and presented as percentage of the initial activity

^a,b^Substrate (6 mM) plus catalase re-dosage was done after 2 and 4 days of reaction, respectively

**Fig. 6:**
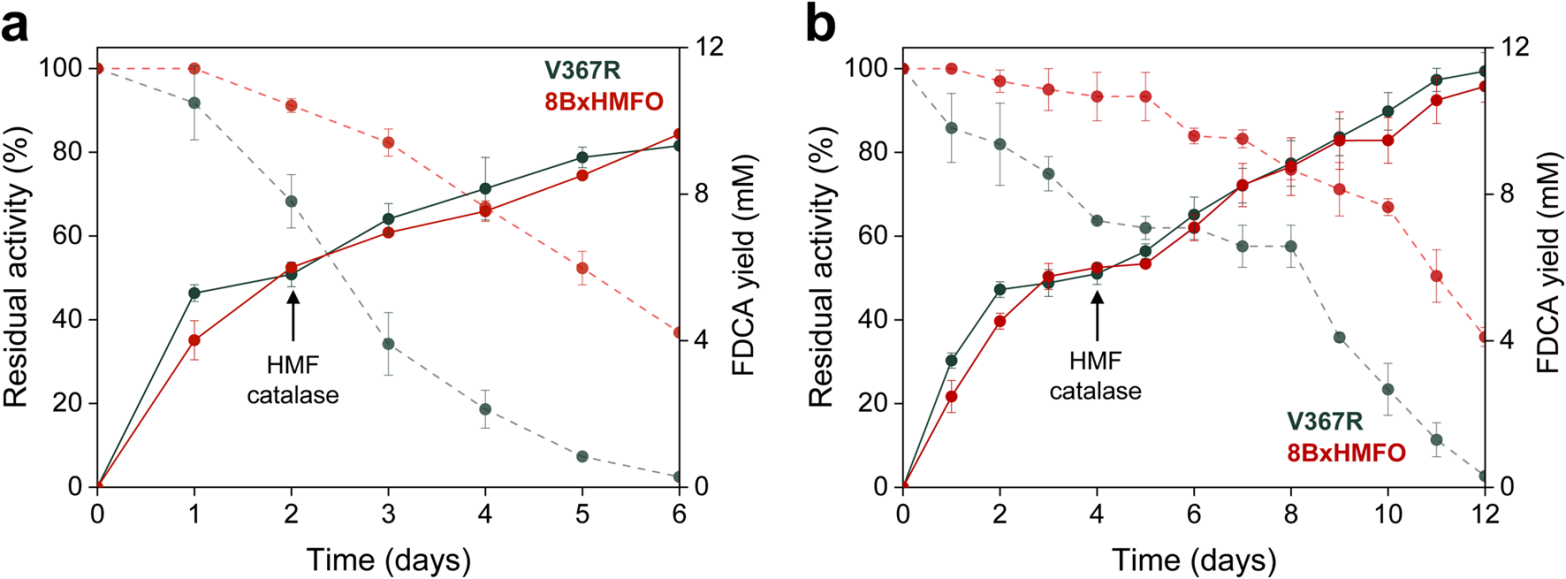
Optimized HMF conversion using two different V367R and 8BxHMFO doses. **a** Reactions with 2.5 µM enzyme, and HMF/catalase re-dosage after 2 days. **b** Higher FDCA production by lowering the enzyme dose (to 1.25 µM), and HMF/catalase re-dosage after 4 days. FDCA yields (lines) and residual enzyme activities (dashes) are shown during HMF (6 mM concentration, plus 6 mM addition after 2 or 4 days) reactions with the V367R (black) and 8BxHMFO (red) variants, in 50 mM Tris/HCl, pH 8.0, in the presence of catalase. FDCA production was quantified by HPLC, and residual activities were measured with vanillyl alcohol, in 50 mM Tris/HCl, pH 7.5. Mean and standard deviation values from triplicate experiments are shown

Therefore, taking advantage of the high residual activity of V367R and 8BxHMFO at the end of reactions with 6 mM HMF, we decided to supplement the reactions with additional 6 mM HMF and catalase excess when the first conversion was completed after 2 days (Fig. 6a). In this way, almost 10 mM FDCA was attained in 6 days reaching total turnover numbers (TTN) over 11,000 (Table 4). As FFCA accumulation under these conditions could result in enzyme inactivation limiting final product concentration, lower enzyme doses (1.25 µM) were assayed (Fig. 6b). Under these conditions, slower production and minor accumulation of FFCA was expected, resulting in more active enzyme being able to complete the conversion till FDCA. With this amount of enzyme, a second addition of substrate and catalase was applied after 4 days of reaction, when no more conversion was observed. In this way, almost 12 mM FDCA was obtained after 12 days, before enzyme inactivation, and TTN values of 26,000 and 27,000 were reached for the 8BxHMFO and V367R variants, respectively (Table 4). Therefore, taking advantage from the high stability of both variants under optimized conditions, we were able to double the FDCA yields obtained by re-dosing substrate to overcome the inhibition by the intermediate FFCA. By applying these reaction conditions, FDCA yields ≥ 90% are attained facilitating downstream procedures to obtain pure FDCA. However, further reaction upscaling is necessary for more realistic evaluation of product purification under industrially-relevant conditions.

## Conclusions

Biocatalysts for industrial processes must fulfill specific features to ensure the feasibility and applicability of the resulting bioprocess. Aspects such as enzyme stability, cofactor requirement, reaction conditions and substrate range have to be optimized for industrial application [41]. Here, we have identified several limitations of HMFO that can prevent an efficient production of FDCA from HMF, such as the low stability of the enzyme under high concentrations of H_2_O_2_, and its strong inhibition by the FFCA intermediate. However, the above limitations in the enzymatic production of FDCA from HMF can be overcome with the addition of catalase to the reaction mixture and controlling the dose of substrate, respectively. Other aspects such as O_2_ diffusion into the reaction medium are also important for a further optimization of these processes. The above findings should be taken into account for the use of HMFO variants in an industrial context. They will also pave the way for the characterization of other HMFOs as well as in the development of new variants that enable a more efficient production of FDCA from HMF.

## Materials and methods

### Chemicals

HMF was kindly provided by AVA Biochem. DFF, FDCA, FAD, catalase, HRP, vanillyl alcohol was purchased from Sigma-Aldrich (St. Louis, MO, USA). FFCA was purchased from TCI America (Portland, OR, USA). AmplexRed® was obtained from Invitrogen (Walthem, MA, USA).

### Genes, plasmid and site-directed mutagenesis

The gene encoding HMFO from *Methylovorus* sp MP688 (NCBI accession number WP_013440946) was synthesized by ATG biosynthetic (Merzhausen, Germany) and then subcloned into the pET23b(+) plasmid (Novagen). Simple variants of HMFO were performed by whole plasmid PCR using the pET23b-HMFO plasmid as a template. Forward and reverse primers were designed complementary to opposite strands of the DNA region containing the desired mutation. The sequence of the forward synthetic primers (substituted nucleotides are in bold and the triplet containing the mutation is underlined) were 5'-GCA AGC GCT **CGT** TTC TGG GTG AAC AAG C-3' for V367R and 5'-C GTC GGC GGT GTT T**TT** CAT GCG AG-3' for W466F. For V367R/W466F, W466F primers were employed using variant V367R as template. Template DNA was cleaved with *Dpn*I (Roche). *E. coli* DH5α cells were transformed with the plasmids through thermal shock. Plasmid purification from *E. coli* DH5α cultures in LB-ampicillin 100 µg/mL was carried out using High Pure Plasmid Isolation Kit (Roche). The octuple HMFO variant (8BxHMFO) containing mutations I73V, H74Y, G356K, V367R, T414K, A419Y, A435E and W466F was synthesized by ATG biosynthetic (Merzhausen, Germany) and subcloned into the pET23b(+) plasmid (Novagen). Introduction of mutations was confirmed by sequencing.

### Enzyme production and purification of HMFOs

For recombinant protein expression, the constructed plasmids were transformed into *E. coli* BL21(DE3)pLysS cells. Overnight cultured cells were diluted 1:30 in 1 L of LB medium containing 100 µg/mL ampicillin and 34 µg/mL chloramphenicol, and grown at 37 °C and 200 rpm until an OD_500nm_ of 1.0 was reached. Cells were induced with 0.1 mM isopropyl β-d-1-thiogalactopyranoside for 72 h at 16 °C and 150 rpm. Cells were harvested by centrifugation at 7000 rpm for 5 min at 4 °C and frozen for the expression of lysozyme. Bacterial pellets were resuspended in lysis buffer (50 mM Tris/HCl, pH 8.0, supplemented with 10 mM EDTA and 5 mM dithiothreitol) and treated with 0.1 mg/mL of DNase (Roche) at 4 °C for 30 min. The cell extracts were obtained after cell disruption through sonication (10 cycles of 1 min at 4 °C), centrifugation at 13,000 rpm and 4 °C for 30 min, and ultracentrifugation of supernatant at 30,000 rpm and 4 °C for 1 h to eliminate insoluble debris. The soluble fraction obtained was preserved at − 20 °C until its purification.

Native HMFO and variants were purified following the same protocol previously described for HMFO WT [24]. Two consecutive anionic-exchange chromatographic steps, first with a Resource Q 6-mL (GE Healthcare) column and then with Mono Q 5/50 GL 1-mL column were used to obtain pure fractions of the enzymes. The purification process was followed by analyzing each fraction in a SDS-PAGE 12% (*v*/*v*) polyacrylamide separation gel (Additional file 1: Fig. S7).

### Spectral properties of HMFOs

The UV–visible spectra of the purified proteins were recorded between 250 and 700 nm in a Cary4000 spectrophotometer. The spectra of the folded enzymes and the free flavin, obtained by heat treatment of the enzyme (95 °C for 5 min) and centrifugation for 45 min, were recorded to calculate the extinction coefficient at the band-I of the flavin, using the known extinction constant for FAD (ε_450nm_ = 11,300 mM^−1^ cm^−1^) [42] (Additional file 1: Table S3). The calculated extinction coefficient of each variant was used to estimate the protein concentrations.

### pH and H_2_O_2_ stability of native HMFO and variants

pH stability was estimated by incubating the purified enzymes at different pH (6–9) in Britton-Robinson buffer. To determine stability to H_2_O_2_, purified enzymes were incubated in the presence of 0 to 30 mM H_2_O_2_ in 50 mM Tris/HCl, pH 7.5. Samples were incubated at 25 °C and residual activities were measured at 0, 24, 48 and 72 h by oxidation of 3 mM vanillyl alcohol in 50 mM Tris/HCl, pH 7.5, at 25 °C. For each enzyme, the highest activity obtained at the different times and pH or H_2_O_2_ conditions was taken as 100%, and the percentages of residual activity of the remaining measures were referred to this value.

### FDCA production by HMFOs

Effect of pH on HMF and FFCA oxidation was determined by analyzing FDCA yields after incubating each enzyme (2.5 µM) with HMF or FFCA (1.5 mM) in 50 mM NaPi, pH 6.5, and in 50 mM Tris/HCl, pH 7.5, 8.0 and 9.0. Effect of H_2_O_2_ on FDCA production was analyzed by incubating HMFO WT (2.5 µM) with HMF (1.5 mM) in presence of different concentrations of H_2_O_2_. FDCA:H_2_O_2_ stoichiometry was evaluated by quantifying FDCA by HPLC (see below) and H_2_O_2_ using AmplexRed®/HRP. Effect of oxygen was studied by analyzing FDCA yields after incubation of the enzymes (2.5 µM) with HMF (6 mM) and catalase excess (10–25 U/mL) and bubbling the reactions with 1.22 mM of O_2_. Effect of cofactor addition was assayed by incubating the enzymes (2.5 µM) with 6 mM HMF (6 mM) with and without the addition of FAD (20 μM).

All reactions (unless indicated) were performed in 50 mM NaPi, pH 6.5, for HMFO WT and 50 mM Tris/HCl, pH 8.0, for HMFO variants, at 28 °C under continuous shaking (180 rpm). In all cases, solutions were treated in the same conditions but in the absence of enzyme as negative controls.

Residual activity of HMFOs along the reactions was measured just after taking aliquots at different times (usually 0, 4, 24 and 48 h or every 24 h in longer reactions), before addition of HCl, by monitoring their activity against vanillyl alcohol. The activity decay as a function of time was calculated from Eq. 1 allowing the estimation of the half-life (Eq. 2):

1 $$HMFOactivity\; \left( \% \right) = HMFO act_{0} \, \cdot e^{ - \lambda t}$$

2 $$t_{1/2} = \frac{ln\left( 2 \right)}{\lambda }$$

where *λ* is the activity decay constant, and t_1/2_ is the enzyme half-life.

### Product identification

After substrate conversion, 150 µL aliquots were taken at different times (usually 0, 4, 24 and 48 h or every 24 h in longer reactions) and reactions were stopped by adding 1 M HCl up to pH 2–3. Furfural oxidation was analyzed by HPLC using an ion-exchange SUPELCOGEL C-610H column (300 × 7.8 mm, 9 µm particle size, SUPELCO). Compounds were eluted using 5 mM H_2_SO_4_ as mobile phase at a flow rate of 0.6 mL/min at 30 °C and detection was done at 264 nm. The retention times of FDCA, HMFCA, FFCA, HMF and DFF were 21, 27, 30, 42 and 52 min, respectively (Additional file 1: Fig. S8). Calibration curves were made with 0.05, 0.1, 0.2, 0.4, 0.75 and 1.5 mM solutions of each component that might be present during the reaction. H_2_O_2_ quantification was performed using a peroxidase coupled assay before the addition of HCl. In this assay, HRP (6 U/mL final concentration) reacts with AmplexRed® reagent (75 ng/mL final concentration) in presence of H_2_O_2_ producing a pink product (resorufin; ε_563nm_ = 52,000 M^−1^ cm^−1^). Quantification was performed using a calibration curve with known concentrations of H_2_O_2_. The standard deviations were always ≤ 5% of the mean values obtained.

### Kinetic analysis

Steady-state kinetic parameters for HMF and DFF oxidation by HMFOs were calculated by monitoring the production of H_2_O_2_ during oxidation of the different substrates using a HRP-coupled assay with AmplexRed® as final substrate in 50 mM Tris/HCl, pH 7.0, as previously described [24]. The oxidation of FFCA was measured in end-time mode by incubating different concentrations of FFCA (0.4–25 mM) with the enzymes (0.3–1.5 µM, depending on the enzyme) supplemented with catalase excess (10–25 U/mL) at 25 °C in 50 mM NaPi, pH 6.0–6.5, or 50 mM Tris/HCl, pH 7.0–9.0, under continuous shaking. Reactions were stopped after 48 h for WT HMFO or at 30 min for HMFO variants, and the products were quantified by HPLC as described above. The rate of FDCA or H_2_O_2_ formation (*v*/[Ez]) was estimated as the product formed (µmol) *per* enzyme concentration (µmol) *per* time of reaction (resulting in min^−1^ units).

In all cases, kinetic parameters were determined by fitting the initial reaction rates at different alcohol or aldehyde concentrations to the Michaelis–Menten equation (Eq. 3) using Origin software.

3 $$\frac{{v_{0} }}{\left[ E \right]} = \frac{{k_{cat} \left[ S \right]}}{{K_{m} + \left[ S \right]}}$$

where *k*_cat_ is the catalytic constant, and *K*_m_ is the Michaelis–Menten constant.

When apparent kinetic parameters could not be estimated, the linear relation between the rates (min^−1^) and the substrate concentration (mM) was used as a measure of catalytic efficiency (*k*_obs_/[FFCA]).

## Supplementary Information

**Additional file 1: Table S1.** Steady-state kinetic parameters for HMF, DFF and FFCA oxidation. **Table S2.** Effect of FAD on FFCA oxidation. **Table S3.** Spectroscopic properties of the HMFO WT and variants. **Fig. S1.** Effect of H_2_O_2_ on FDCA production from FFCA. **Fig. S2.** Kinetic curves of HMF and DFF oxidation. **Fig. S3** Effect of FFCA on HMFO kinetics and residual activity. **Fig. S4.** Effect of oxygen, FAD and catalase on FDCA production. **Fig. S5.** HMF control reactions without HMFO. **Fig. S6.** Reactions of HMFO variants with different HMF concentrations. **Fig. S7.** SDS-PAGE of purified HMFOs. **Fig. S8.** HPLC separation of HMF-derived furfurals.

## Data Availability

All the data supporting the conclusions of this article are included within the article and its supporting information. Additional information can be provided by the corresponding authors under request.

## Abbreviations

AAO: Aryl-alcohol oxidase
FDCA: 2,5-Furandicarboxylic acid
FFCA: 5-Formylfurancarboxylic acid
GMC: Glucose-methanol-choline oxidase/dehydrogenase (enzyme superfamily)
HMF: 5-Hydroxymethylfurfural
HMFCA: 5-Hydroxymethylfurancarboxylic acid
HMFO: HMF oxidase
HPLC: High-performance liquid chromatography
HRP: Horseradish peroxidase
*k*_cat_: Catalytic constant
*k*_cat_/*K*_m_: Catalytic efficiency
*K*_m_: Michaelis constant
PEF: Poly(ethylene-2,5-furandicarboxylate)
PET: Poly(ethylene-terephthalate)
TTN: Total turnover number
