# Supplementary material for: Optimizing operational parameters for the enzymatic production of furandicarboxylic acid building block
Source: Microb Cell Fact. 2021 Sep 9;20:180. doi: 10.1186/s12934-021-01669-1 (PMC8427864; doi:10.1186/s12934-021-01669-1)
Supplement: Supplementary file 1 — Additional file 1: Table S1. Steady-state kinetic parameters for HMF, DFF and FFCA oxidation. Table S2. Effect of FAD on FFCA oxidation. Table S3. Spectroscopic properties of the HMFO WT and variants. Fig. S1. Effect of H2O2 on FDCA production from FFCA. Fig. S2. Kinetic curves of HMF and DFF oxidation. Fig. S3 Effect of FFCA on HMFO kinetics and residual activity. Fig. S4. Effect of oxygen, FAD and catalase on FDCA production. Fig. S5. HMF control reactions without HMFO. Fig. S6. Reactions of HMFO variants with different HMF concentrations. Fig. S7. SDS-PAGE of purified HMFOs. Fig. S8. HPLC separation of HMF-derived furfurals. [file 12934_2021_1669_MOESM1_ESM.pdf]

SUPPLEMENTARY INFORMATION

Optimizing operational parameters for the enzymatic production of furandicarboxylic acid building block

María Isabel Sánchez-Ruiz , Angel T. Martínez* and Ana Serrano*

This Supplementary Information includes: Steady-state kinetic parameters for HMF, DFF and FFCA oxidation (**Table S1**), Effect of FAD on FFCA oxidation (**Table S2**), Spectroscopic properties of the HMFO WT and variants (**Table S3**), Effect of H_2O_2 on FDCA production from FFCA (**Fig. S1**), Kinetic curves of HMF and DFF oxidation (**Fig. S2**), Effect of FFCA on HMFO kinetics and residual activity (**Fig. S3**), Effect of oxygen, FAD and catalase on FDCA production (**Fig. S4**), HMF control reactions without HMFO (**Fig. S5**), Reactions of HMFO variants with different HMF concentrations (**Fig. S6**), SDS-PAGE of purified HMFOs (**Fig. S7**) HPLC separation of HMF-derived furfurals (**Fig. S8**).

Table S1. Steady-state kinetic parameters [k_{cat} (min^{-1}), K_m (mM), k_{cat}/K_m ($mM^{-1}min^{-1}$)] **for HMF, DFF and FFCA oxidation by HMFO WT and variants**.

		WT	V367R	W466F	V367R/W466F	8BxHMFO
	k_{cat}	82.5 ± 3.2[b]	44.8 ± 1.0	1.4 ± 0.03	3.4 ± 0.2	5.4 ± 0.2
**HMF**[a]	K_m	9.1 ± 0.7[b]	4.2 ± 0.3	6.4 ± 0.5	23.1 ± 2.8	17.1 ± 1.6
	k_{cat}/K_m	4.6 ± 0.4[b]	5.4 ± 0.4	0.11 ± 0.01	0.07 ± 0.01	5.4 ± 0.4
	k_{cat}	6.1 ± 0.2[b]	2.2 ± 0.1	0.3 ± 0.01	1.5 ± 0.04	2.5 ± 0.1
**DFF**[a]	K_m	6.8 ± 0.6[b]	1.1 ± 0.2	1.8 ± 0.3	4.9 ± 0.5	2.7 ± 0.3
	k_{cat}/K_m	0.9 ± 0.1[b]	2.0 ± 0.4	0.16 ± 0.03	0.31 ± 0.03	0.9 ± 0.1
	k_{cat}	0.46 ± 0.02	4.9 ± 0.1	5.4 ± 0.4	9.4 ± 0.2	11.2 ± 0.3
**FFCA**[c]	K_m	2.6 ± 0.3	1.3 ± 0.1	3.7 ± 0.5	0.12 ± 0.02	0.09 ± 0.02
	k_{cat}/K_m	0.18 ± 0.01	3.7 ± 0.3	1.8 ± 0.3	66 ± 7	130 ± 16

[a] Kinetics in 50 mM Tris/HCl, pH 7.0, for the HMFO variants, were measured by H_2O_2 release using the AmplexRed®/HRP assay. Mean and standard deviation values are shown.

[b] Taken from Viñambres M, Espada M, Martínez AT, Serrano A. Screening and evaluation of new hydroxymethylfurfural oxidases for furandicarboxylic acid production. Appl Environ Microbiol. 2020, 86, 16, e00842-20.

[c] Kinetics in 50 mM NaPi, pH 6.5, for HMFO WT and 50 mM Tris/HCl, pH 8.0, for the HMFO variants, were measured by HPLC. Mean and standard deviation values are shown.

* Corresponding authors: anaserra1979@gmail.com and atmartinez@cib.csic.es
Centro de Investigaciones Biológicas "Margarita Salas" (CIB), CSIC, Ramiro de Maeztu 9, E-28040 Madrid, Spain

Table S2. Effect of FAD on FFCA oxidation as shown by apparent catalytic efficiencies (k_{cat}/K_m, $mM^{-1}min^{-1}$) of the reactions with HMFO WT and variants.

FAD	WT	V367R	W466F	V367R/W466F	8BxHMFO
–	0.18 ± 0.02	3.71 ± 0.29	1.84 ± 0.32	66.3 ± 10.3	130.4 ± 28.3
+	0.16 ± 0.02	3.66 ± 0.35	1.65 ± 0.24	77.2 ± 12.8	123.3 ± 14.6

Mean and standard deviation values are shown.

Table S3. Spectroscopic properties of the HMFO WT and variants.[a]

	λ band II (nm)	λ band I (nm)	ε band I (M^{-1} cm^{-1})	$A_{278}/A_{band I}$
**WT**	387	457	10,340	10.7
**V367R**	387	457	9317	10.7
**W466F**	380	449	13,654	9.2
**V367R/W466F**	380	449	11,537	10.4
**8BxHMFO**	383	452	12,047	10.8

[a] Visible spectra were recorded in 50 mM Tris/HCl, pH 7.0.

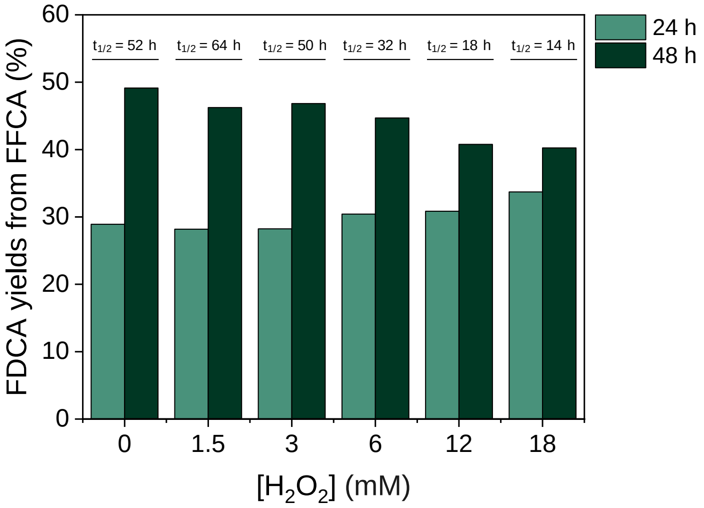

Fig. S1. Effect of H_2O_2 on FDCA production from FFCA. FDCA yields in 48-h reactions of FFCA (1.5 mM) and HMFO WT (2.5 µM) —in 50 mM NaPi pH 6.5, at 28ºC— in the presence of different amounts (0 – 18 mM) of added H_2O_2 (the enzyme half-life, $t_{1/2}$, is indicated for each condition).

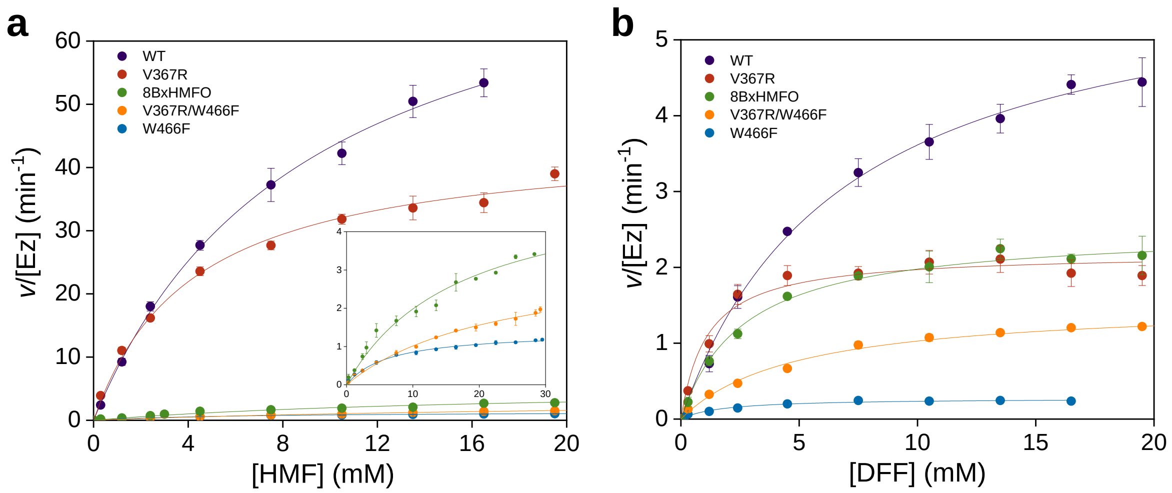

Fig. S2. Kinetics curves of HMF (a) and DFF (b) oxidation by HMFO WT and variants. Steady-state kinetic oxidation was measured by following the H_2O_2 produced during 1 min using a coupled assay with AmplexRed®/HRP. Mean and standard deviation values are shown.

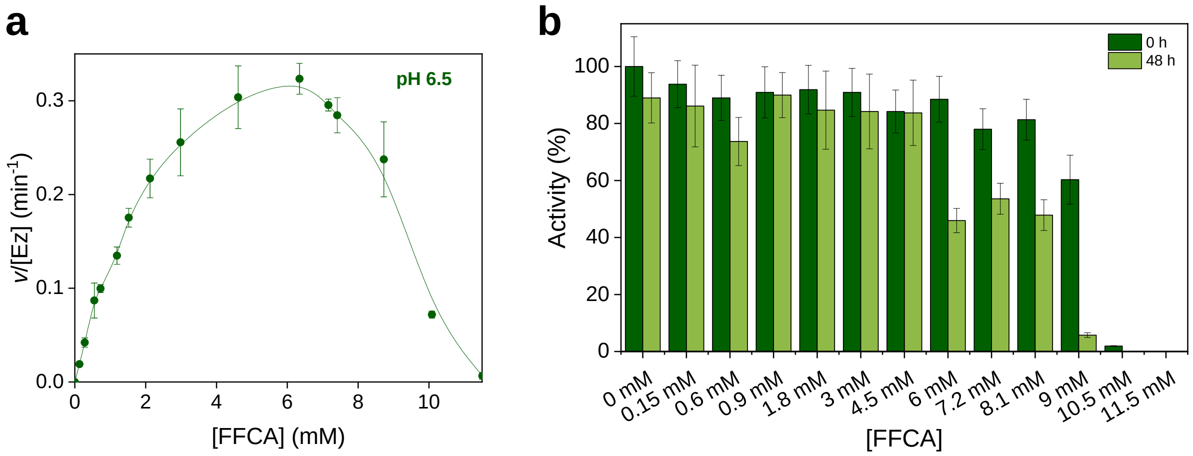

Fig. S3. Effect of FFCA on HMFO kinetics and residual activity. a) Steady-state kinetics curve of FFCA oxidation by HMFO WT at pH 6.5. **b**) Residual activity of the enzyme during FFCA oxidation measured (with vanillyl alcohol) just in the moment of adding the enzyme (t = 0 h) and before the reaction is stopped (t = 48 h). The activity at 0 h in absence of FFCA was taken as 100%, and the percentages of activity at the different FFCA concentrations shown in **b** were calculated according to this maximal value. Mean and standard deviation values are shown.

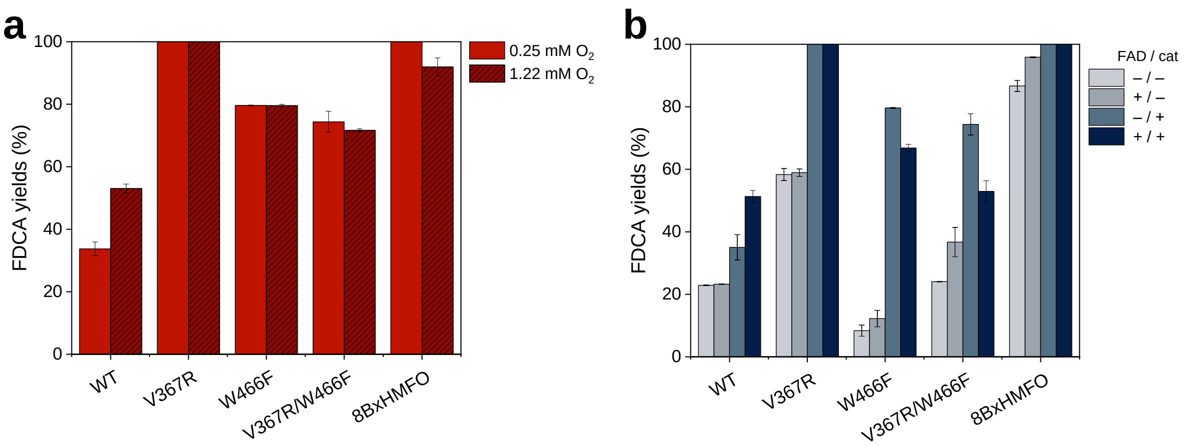

Fig. S4. Effect of oxygen, FAD and catalase on FDCA production. **a**) FDCA yields obtained at estimated 0.25 mM and 1.22 mM O_2 concentrations (obtained by bubbling the reaction with pure O_2 and air, respectively). **b**) FDCA yields in absence (–) and presence (+) of 20 µM FAD or/and catalase excess. Reactions (48 h) between 6 mM HMF and 2.5 µM enzyme were performed in 50 mM NaPi, pH 6.5, for HMFO WT and in 50 mM Tris/HCl, pH 8.0, for the HMFO variants, at 28ºC, and analyzed by HPLC. Mean and standard deviation values are shown.

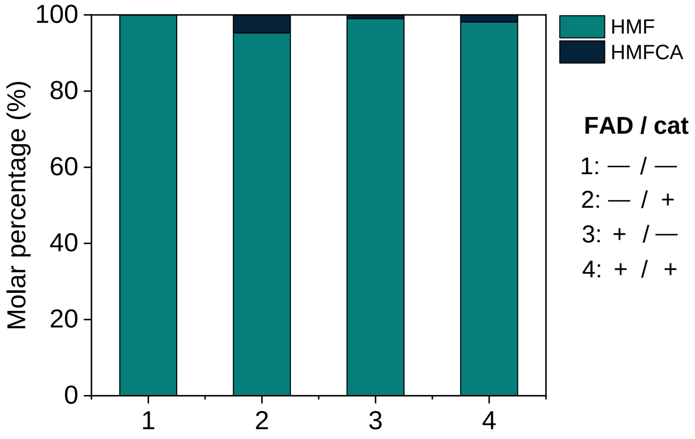

Fig. S5. HMF control reactions without HMFO. Molar percentages of furfural compounds after 48-h incubation of HMF (1.5 mM) in 50 mM sodium phosphate, pH 6.0, at 28ºC in presence (+) or absence (–) of catalase and/or FAD (20 µM), and absence of HMFO.

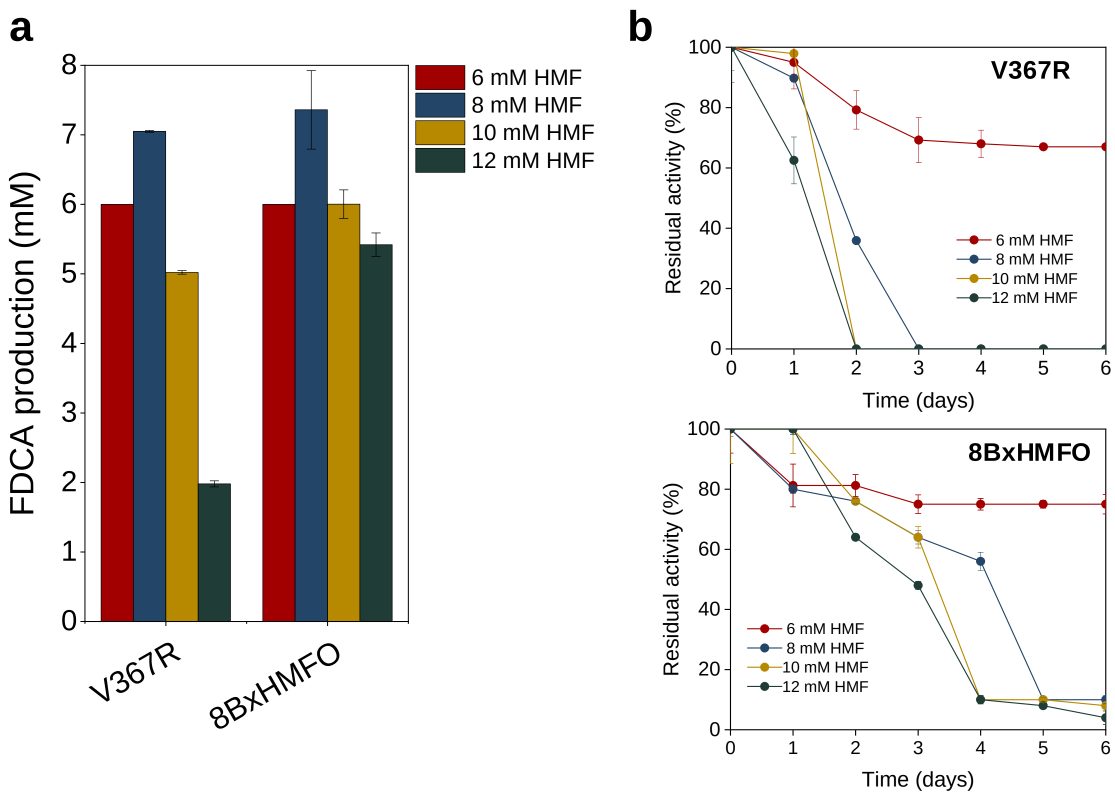

Fig. S6. Reaction of HMFO variants with different HMF concentrations. **a**) FDCA yields by the V367R and 8BxHMFO variants with 6 – 12 mM HMF. **b**) Residual activities of both variants during HMF (6 – 12 mM) conversion. Enzymes (2.5 µM) were incubated with HMF at the indicated concentration in 50 mM Tris/HCl, pH 8.0, in presence of catalase excess, for 6 days at 28ºC under continuous shaking. Residual activities were measured with vanillyl alcohol in 50 mM Tris/HCl, pH 7.5. Mean and standard deviation values are shown.

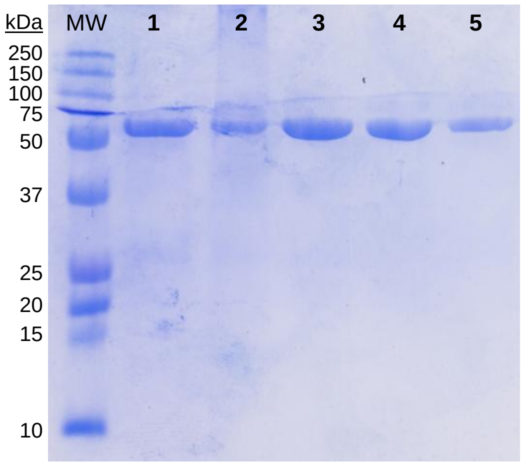

Fig. S7. SDS–PAGE of purified HMFOs. Molecular-mass markers (*MW*) and purified HMFO WT (*lane 1*), V367R (*lane 2*), W466F (*lane 3*), V367R/W466F (*lane 4*) and 8BxHMFO (*lane 5*) from successive Resource Q and Mono Q chromatographic steps.

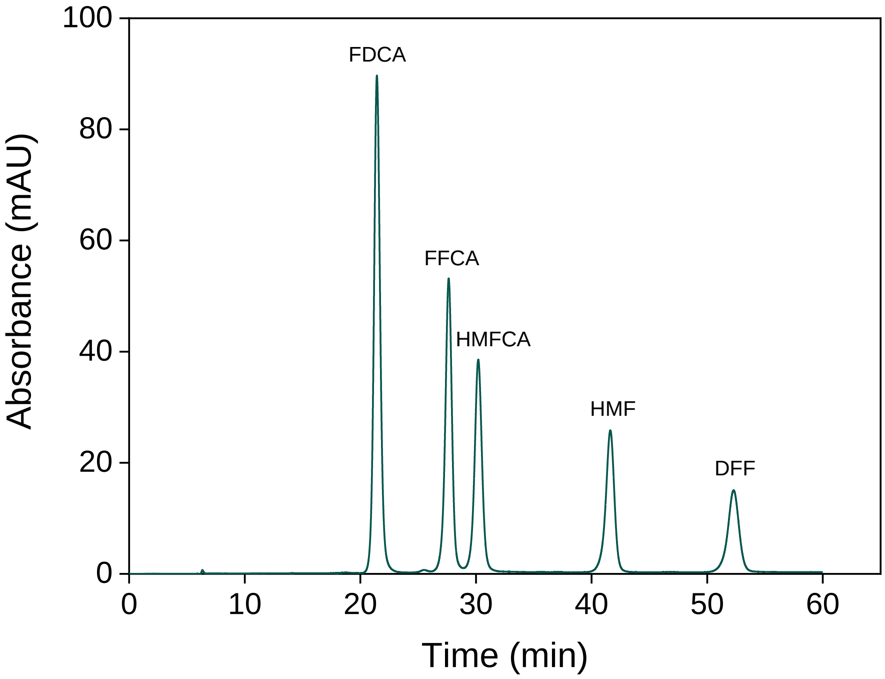

Fig. S8. HPLC separation of HMF-derived furfurals. Furfural standards (0.75 mM) were eluted with 5 mM H_2SO_4 at a flow-rate of 0.6 mL/min at 30ºC, using an ion-exchange SUPELCOGEL C-610H column, and detected at 264 nm. Retention times of FDCA, HMFCA, FFCA, HMF and DFF were 21, 27, 30, 42 and 52 min, respectively.
